# Accelerometry-Enhanced Magnetic Sensor for Intra-Oral Continuous Jaw Motion Tracking †

**DOI:** 10.3390/s21041409

**Published:** 2021-02-18

**Authors:** Mantas Jucevičius, Rimantas Ožiūnas, Mindaugas Mažeika, Vaidotas Marozas, Darius Jegelevičius

**Affiliations:** 1 Biomedical Engineering Institute, Kaunas University of Technology, K. Baršausko g. 59, LT-51423 Kaunas, Lithuania; mindaugas.mazeika@ktu.lt (M.M.); vaidotas.marozas@ktu.lt (V.M.); darius.jegelevicius@ktu.lt (D.J.); 2 Investigo, UAB, Draugystės g. 17-1, LT-51229 Kaunas, Lithuania; info@investigo.lt; 3 Faculty of Electrical and Electronics Engineering, Kaunas University of Technology, Studentų g. 50, LT-51368 Kaunas, Lithuania

**Keywords:** 3D jaw tracker, mastication, trajectory, bruxism evaluation

## Abstract

Currently available jaw motion tracking methods require large accessories mounted on a patient and are utilized in controlled environments, for short-time examinations only. In some cases, especially in the evaluation of bruxism, a non-restrictive, 24-h jaw tracking method is needed. Bruxism oriented, electromyography (EMG)-based devices and sensor-enhanced occlusal splints are able to continuously detect masticatory activity but are uninformative in regards to movement trajectories and kinematics. This study explores a possibility to use a permanent magnet and a 3-axial magnetometer to track the mandible’s spatial position in relation to the maxilla. An algorithm for determining the sensor’s coordinates from magnetic field values was developed, and it was verified via analytical and finite element modeling and by using a 3D positioning system. Coordinates of the cubic test trajectory (a = 10 mm) were determined with root-mean-square error (RMSE) of 0.328±0.005 mm. Possibility for teeth impact detection by accelerometry was verified. Test on a 6 degrees-of-freedom (DOF), hexapod-based jaw motion simulator moving at natural speed confirmed the system’s ability to simultaneously detect jaw position and the impacts of teeth. Small size of MEMS sensors is suitable for a wearable intra-oral system that could allow visualization of continuous jaw movement in 3D models and could enable new research on parafunctional jaw activities.

## 1. Introduction

An ergonomic method for continuous jaw position and activity tracking could provide unprecedented sets of data for detecting, evaluating and researching bruxism. Moreover, it could be used in dental wear experiments for prosthetics’ development, chewing efficiency evaluation, sleep apnea and dysphagia research.

Currently, several methods are available for 3D jaw position tracking. Most significant ones are ultrasonic KaVo Arcus Digma (KaVo Kerr, Brea, CA, USA), optical Modjaw (ModJaw, France), electromagnetic JT-3D (BioResearch Associates Inc., Milwaukee, WI, USA) and magnetic K7 CMS (Myotronics, Kent, WA, USA). In addition, optical marker-enhanced cone beam computed tomography (CBCT) clinical scanners such as “Planmeca 4D Jaw Motion” (Planmeca, Helsinki, Finland) are available. These methods are precise and effective in jaw kinematics and condylar movement evaluation. Such devices are perfect for fitting and manufacturing prostheses, as well as for orthognathic pre-op and post-op evaluation. Nevertheless, in all cases, large external appliances need to be mounted, and steadiness is required from the patient, so 24-h continuous examinations are impossible. Moreover, such precision is unnecessary in patient behavior monitoring, where in order to determine frequency and intensity of parafunctional episodes, mostly the type and nature of jaw movement trajectories are of importance, as well as teeth contact detection. In these cases, a priority lies in prolonged, ergonomic monitoring, with opportunity for patients to continue about their routine with as little intervention as possible.

As for continuous and minimally restricting mandibular activity tracking solutions, most developments are made in bruxism monitoring. Bruxism is an increasingly common oral habit consisting of involuntary rhythmic or spasmodic non-functional gnashing, grinding, or clenching of teeth, which may lead to trauma [1]. In adults, the prevalence of frequent bruxism is 8%, and the total prevalence of bruxism, including mild forms, may be up to 31.4% [2]. On natural teeth, wear can cause loss of vertical dimension, aesthetic problems, hypersensitivity to cold and hot, pulpitis, loss of masticatory efficiency, etc. Tooth clenching causes headache, hypertrophy and myalgia on masticatory muscles and disc displacement or degenerative lesions in temporomandibular joint (TMJ) [3]. Bruxism is suggested to cause an excessive load on implant-supported rehabilitations, which may result in implant fracture, bone loss around the implants and subsequent implant failure [4]. Non-instrumental bruxism diagnosis is usually made from self-evaluation of the patient and clinical signs, such as muscle tension, jaw morning stiffness, headache, grinding noises during sleep and signs of dental wear. However, these signs are not always present; therefore, their absence does not necessarily indicate the absence of bruxism [3]. The current standard for diagnosing sleep bruxism uses polysomnography (PSG) with audio-video (AV) recordings [5]. PSG recordings provide the most valuable research diagnostic criteria for sleep bruxism. However, the fact that the technique is time-consuming, cost-intensive and requires overnight hospitalization does not allow it for routine use. Temporomandibular muscle electromyography (EMG) provides key evidence of sleep bruxism. Portable EMG devices for masticatory muscle evaluation are popular instruments for bruxism diagnostics and are characterized by low patient discomfort and cost. However, the EMG method is indirect, it is limited by movement artifacts, skin resistance variation and low signal amplitudes [6]. Another widely researched solution is sensor-enhanced oral splints, with “Bruxane” (Bruxane, Marburg, Germany) being the only one commercially available, which emits vibrational bio-feedback when bitten, and registers bruxism events. The thickness of the occlusal splint is one of the main sources of discomfort for the patients [3]. It also affects the frequency and intensity of bruxism episodes [7], which is a good feature for harm prevention, but also one that discards the measurement. Also worth mentioning is an active electromagnetic resonance based method, JAWAC, which is used in the Brizzy (Nomics, Liege, Belgium) device. It tracks the linear forehead-to-chin distance and is popular for respiration monitoring in sleep apnea detection.

The demand for a novel, portable device for continuous evaluation of stomatognathic function has been increasing in recent years, but there have been no innovative approaches to the problem. To outperform current solutions, the occlusal surface should not be covered, the device should be unobtrusive for a patient and have resistance to moisture and movement artifacts. We raised a hypothesis that a permanent magnet tracking system described in this article could meet such requirements, and it could be enhanced by accelerometric teeth impact detection. Modern MEMS magnetometers and accelerometers are available in integrated ultrasmall and low-energy devices called inertial measurement units (IMU’s) suitable for utilization in intra-oral appliances. Usage of a permanent magnet does not require any electrical contact between mandible and maxilla. It also provides a possibility to use magnetic field equations to determine the relative position of the mandible. It could be used to record jaw movements and dynamic occlusion for a prolonged period, recognize pathological behavior and show the prevalent trajectories of bruxism. Such patient-specific information would be very useful in diagnostics as well as in the process of dental restoration.

## 2. Methodology

### 2.1. Concept

**Figure 1:**
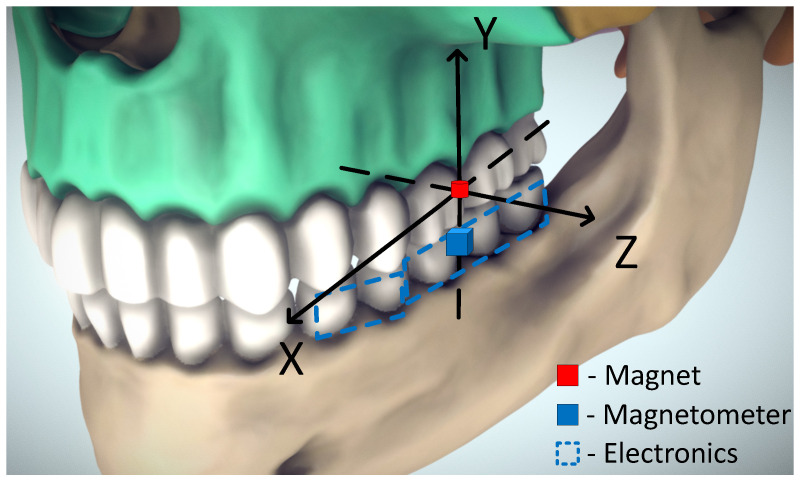
A possible setup for magnetic jaw tracking. Adapted from [9].

The idea of magnetic jaw position tracking is illustrated in Figure 1. It is based on the fact that the magnetic field around a magnetic dipole can be precisely calculated, and every spatial point around it has a unique, non-recurring magnetic field vector [8]. If orientations of a magnet and a 3-axial magnetometer are known, the magnetic field can be used to determine displacement between them. A strong advantage of this method is that no electrical connection between maxilla and mandible is needed. The permanent magnet would be attached to the teeth in the maxilla, and a magnetometer with supporting electronics would be attached to the teeth in the mandible (Figure 1); a vice-versa layout is equally possible.

For this research, magnet of cylindrical shape was chosen, to have radially symmetric field around one axis of rotation. The strongest and smallest available (in retail) magnet was used. It was a NdFeB magnet with a residual magnetism of 1.4 T, resulting in a uniform magnetization magnitude of $M_{0}$ = 1,114,084 A/m directed along rotational axis, length of 2 mm and radius 1 mm.

### 2.2. Theoretical Basis

#### 2.2.1. Finite Element Model

**Figure 2:**
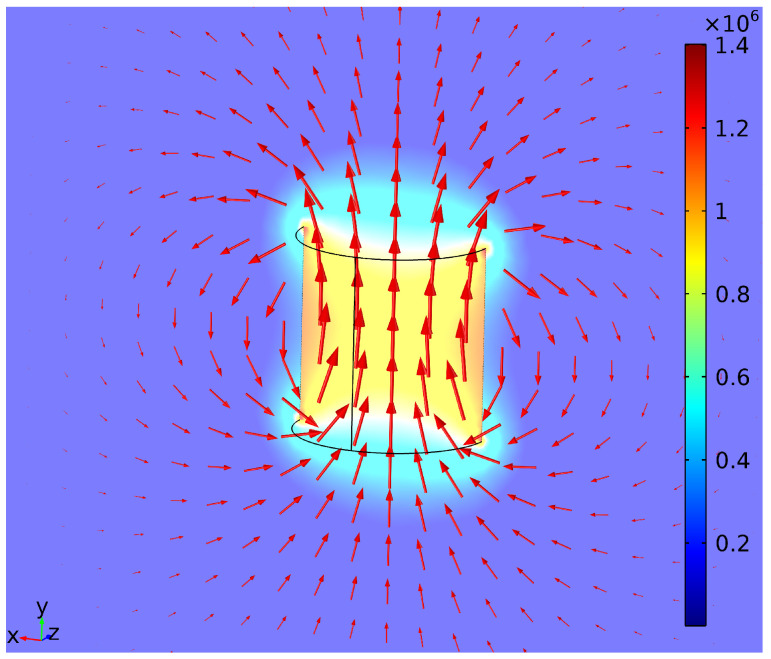
Finite element model of a cylindrical dipole’s magnetic field. Color: magnetic density norm (μT). Arrow (logarithmic): flux density.

In order to assess the potential of the proposed method and better understand the magnetic field surrounding a dipole, finite element modeling of the selected magnet was performed in Comsol Multiphysics 5.1 (COMSOL corporation, Stockholm, Sweden). A snapshot of the model is presented in Figure 2.

#### 2.2.2. Magnetic Position Estimation

Magnetic flux density (*B*) in space surrounding a cylindrical magnetic dipole can be described by a mathematical model described in [10]:

(1) $$B=B_{T}\left(\frac{3(H_{0}\cdot X)X}{R^{5}}-\frac{H_{0}}{R^{3}}\right),$$

where ${\vec{H}}_{0}=\left(m,n,p\right)$ is a normalized unit vector of magnetization direction, describing orientation of the magnet’s magnetism and is equal to (0, 1, 0) for upward orientation of our case. X=((x−a),(y−b),(z−c)) is the location of the magnetometer in relation to the permanent magnet’s location, which is (a,b,c)=(0,0,0). *R* is a normalized *X* vector calculated by (2), and $B_{T}$ is a constant calculated by (3):

(2) $$R=\sqrt{\left({\left(x-a\right)}^{2}+{\left(y-b\right)}^{2}+{\left(z-c\right)}^{2}\right)},$$

(3) $$B_{T}=\left(\mu_{r}\mu_{0}\pi r^{2}LM_{0}\right)/\left(4\pi \right),$$

where $\mu_{r}$ is the relative permeability of the medium (air), $\mu_{0}=4\pi \times 10^{-7}$ (T·m/A) is the magnetic constant, *L* is the length of the magnet (m), *r* is the radius of the magnet (m), and $M_{0}$ is the magnetization magnitude of the magnet (A/m).

Model (1) can be exploded to calculate each axis of the magnetic field vector:

(4) $$Bx=B_{T}\left(\frac{3[m(x-a)+n(y-b)+p(z-c)](x-a)}{R^{5}}-\frac{m}{R^{3}}\right),$$

(5) $$By=B_{T}\left(\frac{3[m(x-a)+n(y-b)+p(z-c)](y-b)}{R^{5}}-\frac{n}{R^{3}}\right),$$

(6) $$Bz=B_{T}\left(\frac{3[m(x-a)+n(y-b)+p(z-c)](z-c)}{R^{5}}-\frac{p}{R^{3}}\right),$$

It is possible to calculate magnetic field values if coordinates and orientations of objects are known. However, a vice-versa task has too many interconnected variables to express coordinates back from *B* values. A Trust Region Reflective algorithm of Least-Square Error (LSE) method was chosen to solve the equations, and an algorithm to determine coordinates from measured *B* values was developed in Matlab (Mathworks Inc., Natick, MA, USA). The algorithm was verified by using finite element modeling (Section 2.2.1).

#### 2.2.3. Mastication Detection

Impacts of teeth can be detected from accelerometry data by using basic signal processing. Vector magnitude calculation, also known as Root Sum Squared (RSS) operation, was sufficient to separate impacts of teeth from other jaw movements by thresholding:

(7) $$RSS\left(X,Y,Z\right)=\sqrt{X^{2}+Y^{2}+Z^{2}},$$

where *X*, *Y* and *Z* are the axial components of the acceleration vector.

### 2.3. Experiment Plan

#### 2.3.1. Effects of BMF

**Figure 3:**
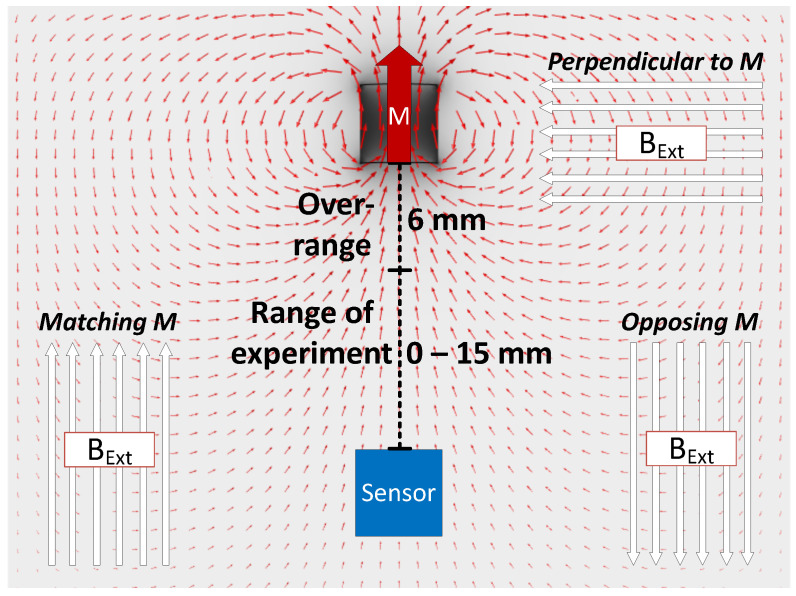
Principle of evaluating the influence of background magnetic field $B_{BMF}$ acting in various directions.

The effects of constant BMF had to be assessed before any positioning experiments. Since the principle of superposition is valid for magnetic fields, all measured values include components of the Earth’s magnetic field. Local BMF values, acquired during the pretest, were subtracted from the measured values. They were replaced by adding theoretical values equal to BMF, of constant size and direction. Three separate simulations were done, with BMF direction matching, opposing and being perpendicular to the magnetic moment of the magnet. Depending on location, Earth’s surface magnetic field is in the range of 25–65 μT [11]. Therefore, maximum value of $B_{BMF}=65$μT was used. The errors induced by BMF of various directions were calculated at different magnet-sensor distances of 15 mm range. Experiments were made on one axis, while other axis’ positions were held at 0. The scheme of the experiment is presented in Figure 3.

#### 2.3.2. Position Tracking

**Figure 4:**
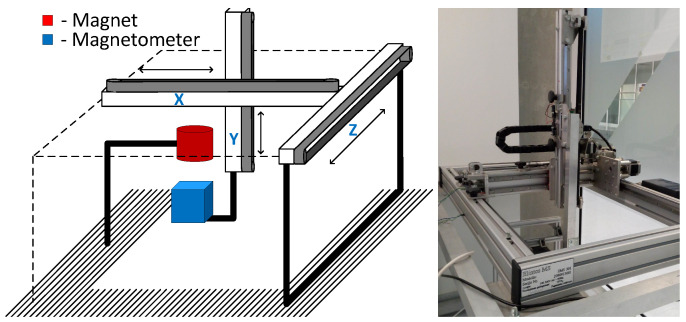
Experimental setup (**left**), and 3D positioning system “EMS 301” (**right**).

Main and reference magnetic field measurements were done using MPU-9250 (TDK InvenSense, San Jose, CA, USA) 9 degrees-of-freedom (DOF) IMU’s containing 3-axial digital magnetometers with dynamic range of ±4912 μT. The dimensions of the whole system on a chip were 3 × 3 mm. The sensors were controlled by the nRF52 Development kit (Nordic Semiconductor, Oslo, Norway). The sensors were calibrated, axially aligned and placed at 35 mm distance from each other. Experiments were performed using a 3D positioning system EMS 301 (Elintos Matavimo Sistemos, Kaunas, Lithuania) with 0.1 mm step positioning resolution, controlled via Matlab. The magnet was mounted in a fixed position, while the sensor was moved by the positioning system. The setup of the experiment is presented in Figure 4.

To assess position tracking error, two test trajectories were used: an easily accessible cubic trajectory with 10 mm border and a real human jaw masticatory cycle trajectory (10 × 7 × 5 mm), sampled from the data presented in [12]. While measuring at each point, every magnetic field value was averaged from 10 consecutive measurements, with the positioning system on hold. In addition, magnetic field values were corrected by local BMF values, acquired during pretest. Errors were calculated between sensor positions determined from magnetic field values and actual sensor positions managed by the positioning system. For each trajectory, full measurements were repeated 10 times. Root-mean-square errors (RMSEs) and standard deviations were used to assess the accuracy of determined trajectories.

#### 2.3.3. Teeth Impact Detection

**Figure 5:**
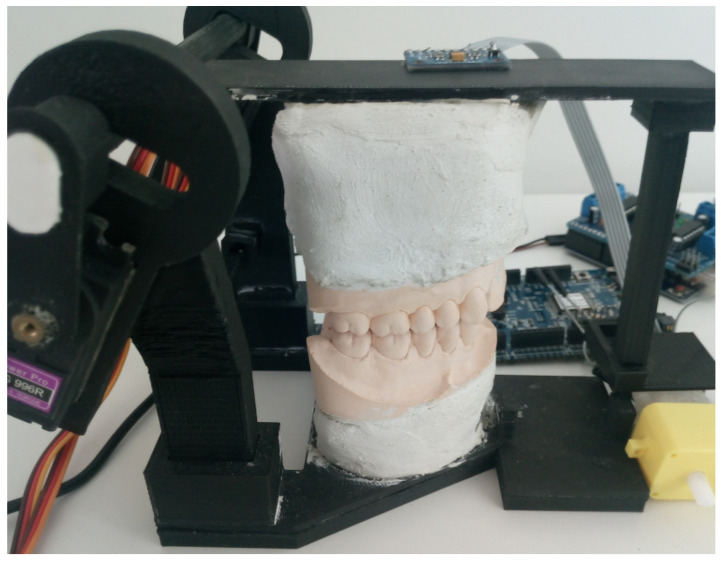
Singlehinge vertical motion articulator with the attached inertial measurement unit (IMU).

A single-hinge vertical articulator was built to simulate vertical jaw motion, and MPU9250-9 DOF IMU containing 3-axial accelerometer was fastened to the moving part. Gypsum teeth were brought to mild contact in 50% of the motion cycles in order to evaluate the performance of the developed algorithm. The setup of the vertical motion articulator experiment is presented in Figure 5. To maximize the mechanical contact, the flat top surface of the articulator was chosen for sensor fixation, and cyanoacrylate glue was used for bonding.

#### 2.3.4. General System Test

**Figure 6:**
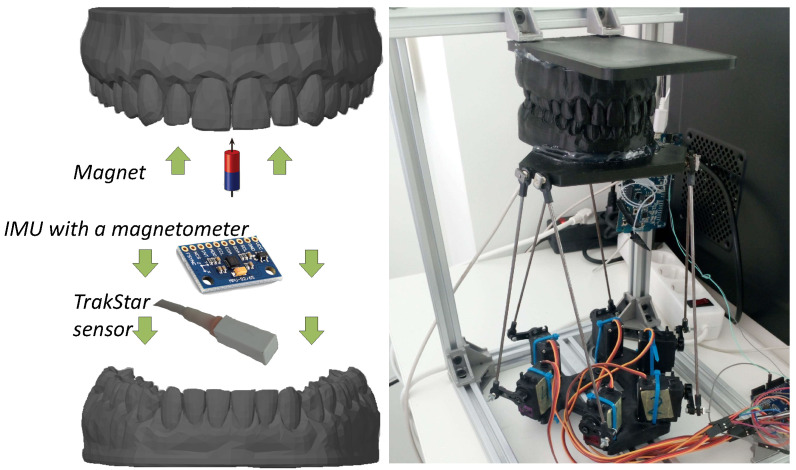
Experimental setup for the general system test using a Stewart platform.

A 6 DOF servo motor driven Stewart platform was built to test the teeth impact detection together with magnetic position tracking, by simulating a masticatory trajectory at a natural speed. The same masticatory curve was used as in position tracking experiment, but due to the unsteadiness of the custom-made, economy-class Stewart platform, the curve was distorted, and each iteration was reproduced slightly differently. Therefore, for a reference position, the electromagnetic tracking system “3D Guidance trakSTAR” (Ascension Technology Corporation, Shelburne, VT, USA) was used. The system works with alternating electromagnetic fields in low radio frequency range, and it neither affects the permanent magnet position measurement nor is affected by it. Data from all sensors were recorded with time-stamps and processed after the experiment, not in real-time. Since the goal of this test is to demonstrate the possibilities of the system, the threshold for impact detection was chosen experimentally, to work on the signals in this specific setup. Models of teeth were 3D-printed from polylactide (PLA). The sensors were fixed onto the mandible with hot glue, and the magnet was fixed to the maxilla with cyanoacrylate glue. The setup of the general system test using the Stewart platform is presented in Figure 6.

In this experiment, the sensor was moving during the measurements; therefore, the data were recorded with a higher frequency of 250 Hz, and the data were smoothed using a moving average filter. Window size of N = 19 was chosen experimentally, by visually evaluating shape and quality of the trajectory line. Compromise had to be made for optimal quality, to preserve real trajectory while smoothing a randomly wavering magnetometer output.

## 3. Results

### 3.1. Finite Element Model

On the basis of the finite element model of the magnet, the beginning of the system’s working range was defined as 6 mm. Since the range of the MPU9250 magnetometer is ±4912 mT, the magnetic field of the magnet met this value at ∼6 mm distance from the dipole’s center.

In space, a magnetic field attenuates following an inverse cube law. It shows that this concept is only suitable for small displacement measurements. In the finite element model, the *B* field of the magnet attenuated to 65 μT (maximum value of the Earth’s surface *B* field [11]) at 17 mm distance from the magnet. At this point, BMF would have a strong influence on the measurement. This indicates a need for BMF compensation.

The LSE method for position estimation was verified using the finite element model. The same test trajectories were used as in latter position tracking experiments. Firstly, in the finite element model, *B* field values were recorded at the coordinates of test trajectories, starting at 6 mm distance from the magnet in vertical (*y*) axis. Then, from the acquired *B* values, new coordinates of the test trajectories were calculated using the LSE approach and compared to originals. Cubic and masticatory trajectory tracking revealed RMSE of 0.231 mm and 0.099 mm, respectively.

### 3.2. Effects of the Background Magnetic Field

**Figure 7:**
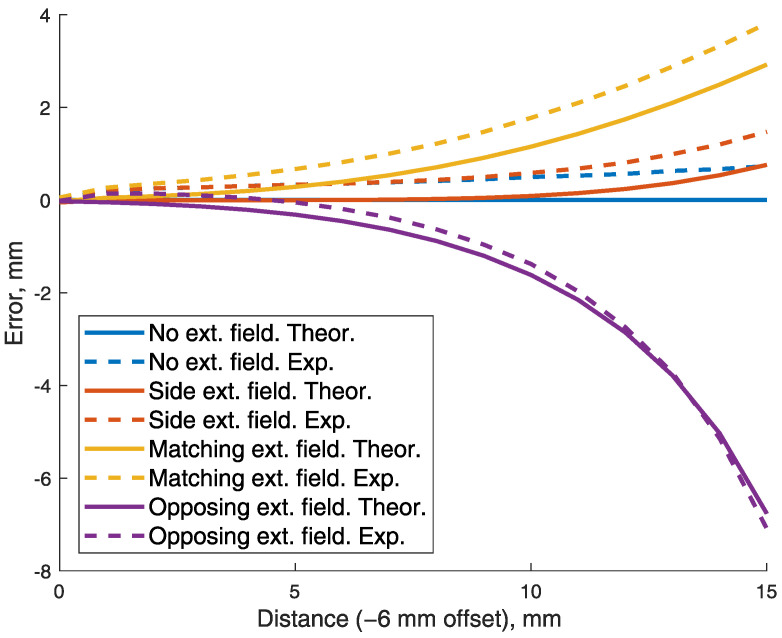
Errors induced by artificial, maximal *B* field of the Earth (65 μT), acting in various directions. Both on theoretical and experimental data.

Errors that would be induced by the 65 μT background magnetic field (BMF) were calculated and presented in Figure 7. By adding values equal to BMF, to both theoretical and experimental data, BMF was simulated in three directions. The graph shows that in close range, the effects of BMF were close to zero. However, BMF had a large influence at 10 mm distance from starting point due to inverse cube law attenuation of the magnetic field. That means increased errors with an open jaw. From a practical point of view, at 10 mm distance from occlusion, teeth are definitely not in contact, so perfect precision is not crucial. Nevertheless, errors of this magnitude significantly decreased the dynamic range of the system. This confirms a need for an active BMF compensation.

### 3.3. Compensating the Background Magnetic Field

**Figure 8:**
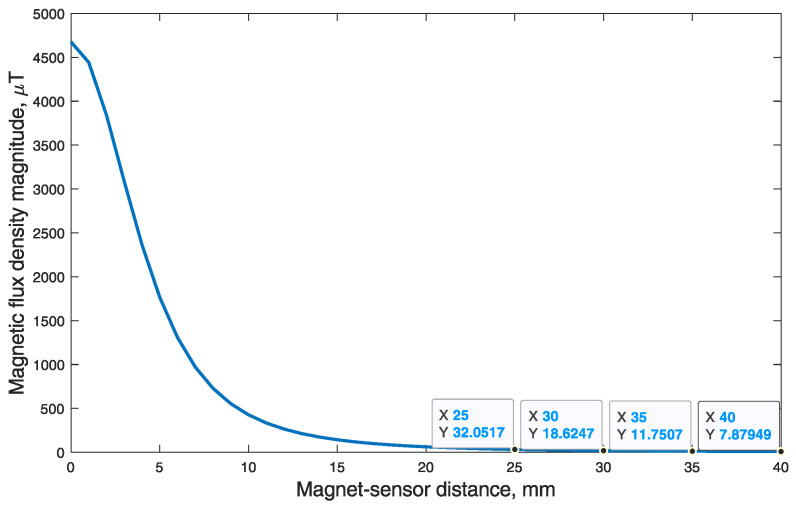
Magnetic flux density magnitude dependency from lateral (z) magnet-sensor distance. Note that vertical distance (y) was constant at 6 mm, which is the zero-point of working range.

The small working range of the method allows for reference magnetometer placement on the same dental arch (but at a distance) as the main sensor. To argue this hypothesis, the magnetic flux density magnitude dependency from a lateral distance (z) was calculated, while vertical distance (y) was kept at 6 mm, which is the zero-point of working range. From calculation results presented in Figure 8, we can see that at 35 mm lateral distance from the magnet, the magnetic flux density was only ∼12 μT. That is significantly lower than the 65 μT maximal magnetic field of the Earth. According to the dental arch dimensions in research by Z. Ahmed [13], in the mandible the dental arch width is 51.27 ± 2.68 mm, while the dental arch length is 33.60 ± 2.94 mm. It should be noted that skull sizes vary depending on gender, ethnicity and genetics of the individual. However, it should be possible to add a reference magnetometer to the proposed system, which would be placed at sufficient distance from the magnet. Therefore, in the following tests a reference magnetometer is used for BMF compensation.

### 3.4. Error of Position Tracking

**Figure 9:**
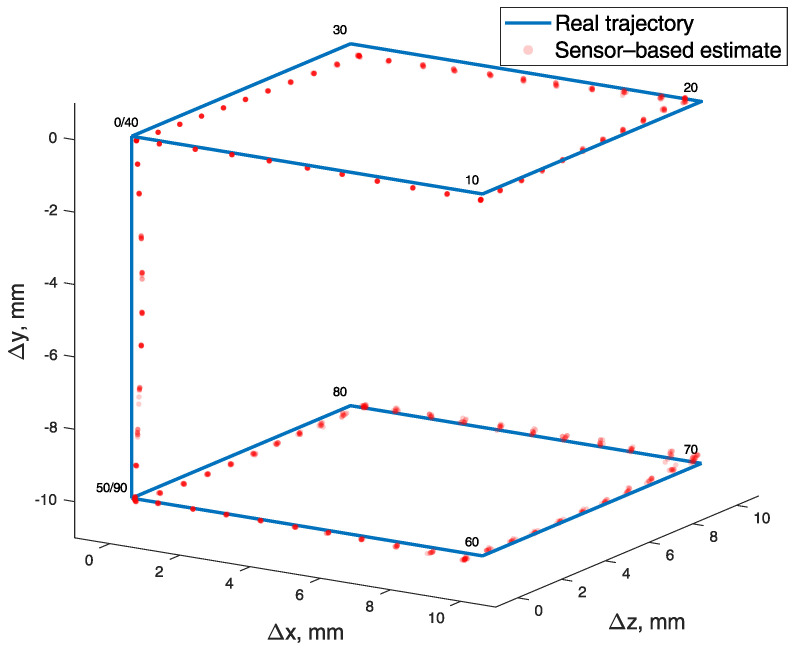
Cubic test trajectory drawn with a 3D positioning system (blue) and determined from magnetic field values (red). Measured points are numbered 0 to 90, and all 10 iterations are represented in transparent markers.

**Figure 10:**
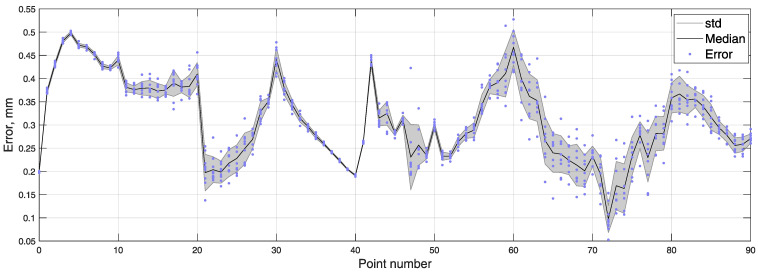
Error of every measured point in 10 iterations of cubic test trajectory, with median and standard deviation.

The position tracking experiments were executed using a 3D positioning system shown in Figure 4. The coordinates of the cubic test trajectory were determined 10 times, resulting in an average RMSE and standard deviation equal to 0.328±0.005 mm. BMF was compensated using a reference magnetometer. Measured points are numbered 0 to 90, and all 10 iterations are represented in transparent markers in Figure 9. To show the error and reproducibility of 10 iterations, the median and standard deviation for each point are presented in Figure 10, with points showing errors of each measurement.

**Figure 11:**
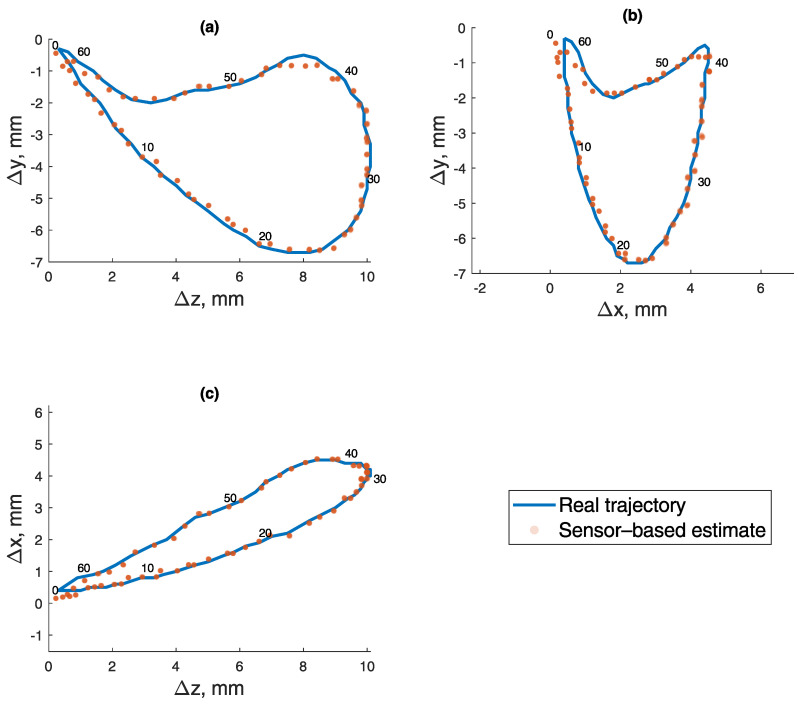
Masticatory test trajectory calculated from magnetic field values. Measured points are numbered 0 to 62, and all 10 iterations are represented in transparent markers. (**a**) Lateral–vertical view (z-y). (**b**) Protrussive–vertical view (x-y). (**c**) Lateral–protrussive view (z-x).

**Figure 12:**
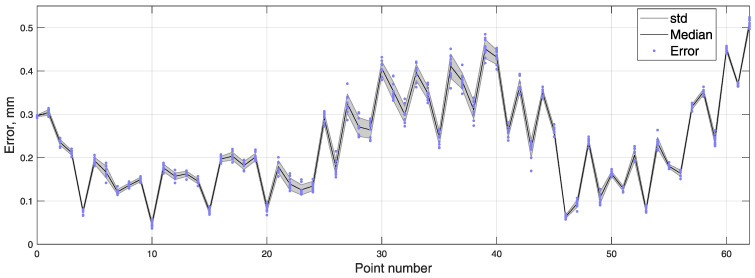
Error of every measured point in 10 iterations of the masticatory test trajectory, with median and standard deviation.

The coordinates of masticatory test trajectory were determined 10 times, resulting in an average RMSE and standard deviation equal to 0.260±0.004 mm. BMF was compensated using a reference magnetometer. Measured points are numbered 0 to 62, and all 10 iterations are represented in transparent markers in Figure 11. To show error and reproducibility of 10 iterations, the median and standard deviation for each point are presented in Figure 12, with points showing errors of each measurement.

### 3.5. Teeth Impact Detection

**Figure 13:**
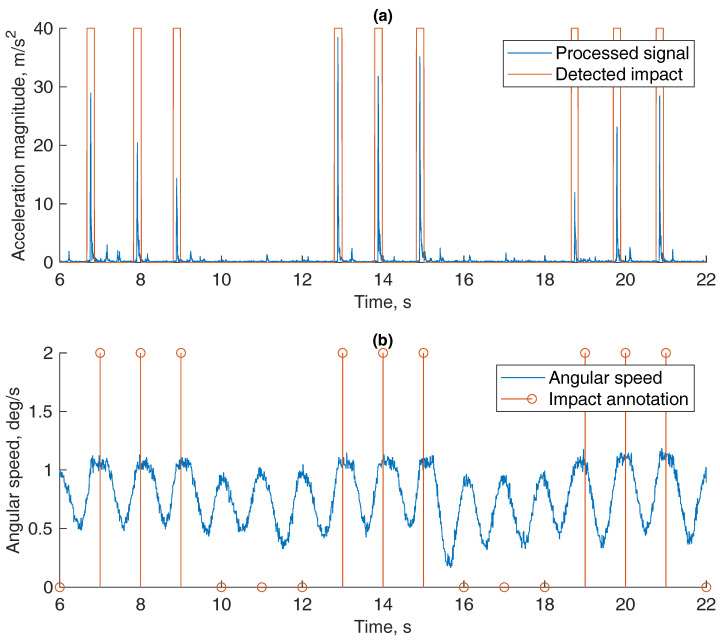
Impact detection in vertical movement. (**a**) Processed accelerometer signal and (**b**) a reference signal of angular velocity with impact annotation.

The signals in this experiment were acquired using a vertical motion articulator shown in Figure 5. In Figure 13, we see a processed acceleration signal with a red line framing detected impacts of teeth. A detection threshold of 5 m/s${}^{2}$ was chosen experimentally. For reference, the angular speed acquired from IMU’s gyroscope data was presented. It showed that motion cycles leading to impacts were of similar speed, rate and nature as the impactless ones. On the same graph, actual impacts are annotated.

### 3.6. General Test of the System

**Figure 14:**
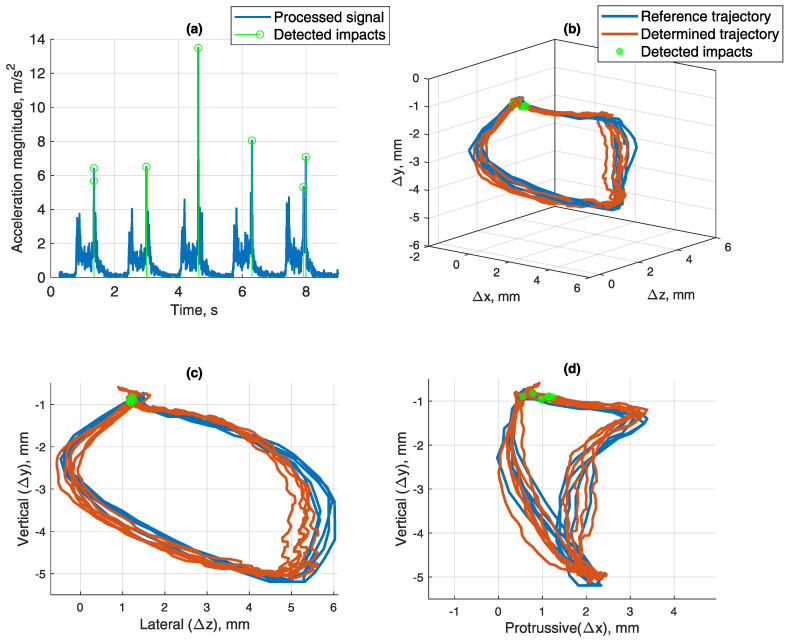
(**a**) Teeth impact detected by an experimentally selected threshold of 5 m/s${}^{2}$, set on acceleration vector’s magnitude value (RSS of X, Y and Z components). (**b**) Masticatory trajectory determined by magnetic position tracking and reference methods, while detected teeth impacts are marked at the points of detection. 3D view. (**c**) Lateral–vertical view (z-y). (**d**) Protrussive–vertical view (x-y).

For the final test, masticatory motions were simulated by our custom-made 6 DOF Stewart platform (hexapod)-based jaw motion simulator shown in Figure 6. In Figure 14a, accelerometry magnitude signal is presented, with detected impacts marked. Detection threshold of 5 m/s${}^{2}$ was chosen experimentally. Two signal peaks can be seen in each iteration; the first one is due to the start of the motion and teeth in jaw model slightly brushing while untouching, and the second one is due to the impact of teeth at the end of the motion. In Figure 14b–d, the locations of detected impacts are marked on the masticatory trajectory, detected by the proposed magnetic position tracking method (red) and a reference position tracking method (blue). As it is seen, highlighted coordinates matched the occlusion segment of the trajectory. During this part, teeth come into contact while closing to occlusion in a lateral-protrussive grinding motion.

## 4. Discussion

Currently, there is no effective way to evaluate 24-h jaw movement. EMG devices and sensor-enhanced occlusal splints are able to continuously detect masticatory activity but are uninformative in regards to movement trajectories and kinematics. Precision 3D jaw position and angle evaluation systems are bulky, expensive and unfit for continuous use. Therefore, a new jaw tracking method was developed and tested that is based on tracking a permanent magnet with a 9 DOF IMU. The possibility to recognize masticatory activity from impacts of teeth using an accelerometer of the same IMU was also investigated.

To the best of our knowledge, precise, small-scale, passive (permanent magnet) tracking has not been used for jaw tracking, although it has been tested for several other biomedical applications. By using a 150 × 150 mm array of 9 magnetic sensors, Dai et al. in 2016 was able to track the position of a magnet with RMSE = 7.48 mm error [14]. In 2017, a magnet was glued to the tip of a tongue for speech rehabilitation research by Sebkhi et al. Three magnetic sensors were used in fixed positions around the mouth, and in a 30 × 30 mm testing area, RMSE < 3 mm was achieved [15]. A promising endoscopic surgical instrument tracking solution was proposed by Song et al. [16]. By using 36 magnetometers in four-wall magnetic tracking space of 0.5 × 0.4 × 0.3 m, they were able to track a dipole magnet with an average error of 0.5 mm and an annular magnet with an average error of 0.003 mm. Predominant multiple-magnetometer solutions expand the dynamic range of the measurements but increase the system’s size, which is crucial for an intra-oral device. We hypothesize that minimizing the number of sensors to a single magnetometer will allow to achieve a comfortable and yet accurate enough method for continuous, long-term (∼24 h) jaw position tracking.

In the experiments, the approach of automatic position and motion simulations was chosen, aiming to prove the effectiveness of proposed method. It was concluded early in the study that the method is susceptible to BMF’s and has subsequent limitations in precision and dynamic range. To address this problem, a reference magnetometer for BMF compensation was used. On the basis of the magnet’s strength-to-distance dependency seen in Figure 8, it was substantiated that the minimum distance of 35 mm would be sufficient for reference BMF measurements to remain unaffected by a permanent magnet’s field. A reference magnetometer located on the opposite side (of the dental arch) from the main sensor was chosen as a remedy for BMF influence. However, this solution has a drawback of increasing the size of the device. With reference BMF correction, the average 10 measurement RMSE and standard deviation was 0.328±0.005 mm for a cubic trajectory (a = 10 mm). With a natural masticatory trajectory (10 × 7 × 5 mm), the 10 measurement average RMSE and standard deviation was 0.260±0.004 mm. The algorithm still managed to calculate sensor position within <1 mm error at 18 mm distance from occlusion, but the optimal working range recommended by the authors is in 15 mm radius from zero position (occlusion). Such range and error level should be sufficient to determine jaw trajectories and thus to distinguish types of masticatory activity. However, signal processing solutions preventing position errors in wide-open jaw cases should be considered. For instance, Kalman filter could be used to maintain consistency in position measurements. This method would allow visualization of continuous jaw movement in 3D models and could enable new research of parafunctional jaw activities.

By using an accelerometer-embedded tooth implant, Cheng-Yuan Li et al. [17] was able to recognize various oral activities with 59.4% success rate using a person-independent classifier, and with 93.8% success rate using a person-dependent classifier. Though bruxism was not mentioned, chewing, drinking, speaking and coughing were successfully categorized. That suggests a possibility to define and recognize mandibular activities using various features extracted by accelerometer signal processing. In our experiments it was confirmed that by using accelerometry data, it is possible to detect impacts of teeth. The existence of on-chip (3 × 3 mm) 9 DOF inertial measurement units, which include a magnetometer, accelerometer and a gyroscope, completely simplifies the task of joining the two methods. Moreover, the methods could benefit from each other, e.g., discard false-positive impacts by checking if a contact is plausible at a particular magnetically estimated position. By simulating the masticatory trajectory at a natural speed with a 6 DOF Stewart platform, it was demonstrated that the system is capable of recording natural jaw movement trajectories and detecting teeth impacts simultaneously.

**Figure 15:**
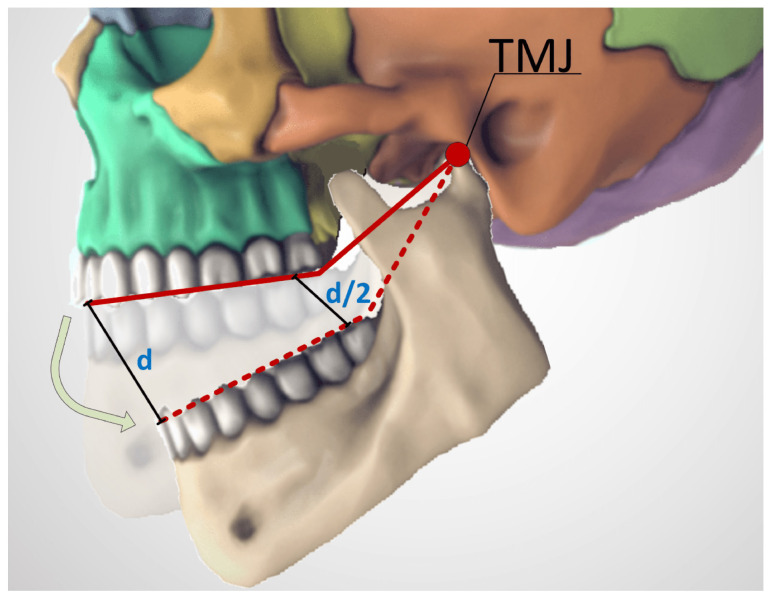
Path difference for sensors mounted at different distances from the temporomandibular joint. For a system with limited dynamic range, a shorter path would allow to record wider jaw movements. Adapted from [9].

The main limitation of the study is a direct dependency of precision on the size of the test trajectory. Therefore, the precision of the experiments would be better with smaller test trajectories, and vice versa. As for the method, using a larger and stronger magnet could increase the system’s precision, dynamic range and make the method more robust and resistant to BMF’s. Magnetometers with higher dynamic range should be used in such cases. However, inverse cube law attenuation of the magnetic field should be taken into consideration. To make a sensible difference, a new magnet should be stronger by at least several orders of magnitude. A stronger magnet would likely be larger and would also increase the minimum distance for reference magnetometer placement, either increasing the size of the system or entirely preventing the use of an active BMF compensation. Regarding sensor positioning, the optimal solution seems to be placing it on the side of the rear molars. With molars being closer to the temporomandibular joint, wider jaw movements can be effectively registered using a system with limited dynamic range, as the sensor movement path is reduced approximately twofold (Figure 15). In addition, molar teeth do not overlap, their sides are aligned and the gear would be much less noticeable there, both visually and in terms of comfort for the patient. In such a case, a reference magnetometer could be mounted on the front incisors or at any point of an opposing side of the dental arch. However, without making a prototype and testing it with live subjects, it is unclear whether it is more ergonomical to mount the device on the mandible or on the maxilla.

In future research, a prototype for a device suitable for mounting on a human jaw needs to be manufactured and tested. After all, no robotic articulator can fully substitute the natural motion and material properties of the jaw. Recorded movements should be reproduced by visualization of a 3D jaw model. Analysis of possible energy sources and methods for data transmission (or storage) would be beneficial. If a much stronger magnet of similar dimensions is acquired, whether active BMF compensation is still possible should be determined. If not, benefits of each improvement should be weighed, and a more advantageous one should be chosen.

In conclusion, the proposed method fills the gap between precision jaw trackers and primitive bruxism detectors. As an intra-oral method, it offers full freedom of movement and does not cover the occlusal surface. In exchange, the working range of the system is limited, and BMF compensation is necessary. However, the working range of the proposed system does cover the jaw displacements of natural movements. During bruxism and mastication, a person does not fully open the jaw. Therefore, a natural masticatory trajectory was used for testing, and it was not close to the limit of the working range. Moreover, we are confident the method’s accuracy is sufficient for particular cause of jaw motion trajectory registration and masticatory activity detection, with average errors standing below 0.5 mm.

## Data Availability

The data presented in this study are openly available in FigShare repository at [doi:10.6084/m9.figshare.13397528].
